# Dietary Pattern and Metabolic Syndrome in Thai Adults

**DOI:** 10.1155/2015/468759

**Published:** 2015-01-29

**Authors:** W. Aekplakorn, W. Satheannoppakao, P. Putwatana, S. Taneepanichskul, P. Kessomboon, V. Chongsuvivatwong, S. Chariyalertsak

**Affiliations:** ^1^Department of Community Medicine, Faculty of Medicine, Ramathibodi Hospital, Mahidol University, Rama VI Road, Ratchathewi, Bangkok 10400, Thailand; ^2^National Health Examination Survey Office, Nonthaburi 11000, Thailand; ^3^Department of Nutrition, Faculty of Public Health, Mahidol University, Bangkok 10400, Thailand; ^4^Ramathibodi School of Nursing, Faculty of Medicine, Ramathibodi Hospital, Mahidol University, Bangkok 10400, Thailand; ^5^College of Public Health Sciences, Chulalongkorn University, Bangkok 10330, Thailand; ^6^Faculty of Medicine, Khon Kaen University, Khon Kaen 40002, Thailand; ^7^Epidemiology Unit, Faculty of Medicine, Prince of Songkla University, Songkhla 90110, Thailand; ^8^Faculty of Medicine, Chiang Mai University, Chiang Mai 50002, Thailand

## Abstract

*Objectives*. To determine the dietary patterns of middle-aged Thais and their association with metabolic syndrome (MetS).* Methods*. The Thai National Health Examination Survey IV data of 5,872 participants aged ≥30–59 years were used. Dietary patterns were obtained by factor analysis and their associations with Mets were examined using multiple logistic regression.* Results*. Three major dietary patterns were identified. The first, meat pattern, was characterized by a high intake of red meat, processed meat, and fried food. The second, healthy pattern, equated to a high intake of beans, vegetables, wheat, and dairy products. The third, high carbohydrate pattern, had a high intake of glutinous rice, fermented fish, chili paste, and bamboo shoots. Respondents with a healthy pattern were more likely to be female, higher educated, and urban residents. The carbohydrate pattern was more common in the northeast and rural areas. Compared with the lowest quartile, the highest quartile of carbohydrate pattern was associated with MetS (adjusted odds ratio: 1.82; 95% CI 1.31, 2.55 in men and 1.60; 95% CI 1.24, 2.08 in women), particularly among those with a low level of leisure time physical activity (LTPA).* Conclusion*. The carbohydrate pattern with low level of LTPA increased the odds of MetS.

## 1. Introduction

Metabolic syndrome (MetS), a cluster of metabolic risk factors, is considered to be an intermediate outcome preceding disability and death from various related diseases such as diabetes mellitus and cardiovascular diseases (CVD) [1]. Dietary pattern is an important factor associated with components of MetS such as obesity, diabetes, dyslipidemia, hypertension, and subsequently CVD [2, 3]. Several studies have demonstrated that an unhealthy dietary pattern is associated with increased CVD risk factors, and a healthy diet is linked to decreased risk [2, 4]. Dietary patterns with red meat and processed meat have been reported to be associated with metabolic factors and CVD, whereas a Mediterranean diet is beneficial to metabolic factors [2, 3].

Similar to other countries in Asia, rice, a carbohydrate-rich source, is a staple food among Thais, and glutinous rice, in particular, is common in the northeast region of Thailand. In Asian populations, studies have reported the association between a carbohydrate-rich dietary pattern and metabolic risk, for instance, in Korea and Japan [5, 6]. A diet high in carbohydrate was reported to be associated with dyslipidemia, diabetes, and MetS in Korean adults [7, 8], whereas a western dietary pattern has been shown to be associated with dyslipidemia in Japanese adults [9]. In addition, a healthy dietary pattern combined with a physically active lifestyle has been reported to reduce the risk of CVD and related risk factors [10]. A study in Korea also reported that the risk of MetS associated with carbohydrate intake was dependent on BMI [5].

The prevalence of metabolic risk factors in developing countries, including Thailand, has increased in the past two decades. Previous studies have shown variation in the distribution of metabolic risk factors by geographic region and area of residence in the Thai population [11–14]. However, studies describing the dietary patterns of Thais are scarce. It is less clear whether dietary patterns play a role in the differences in metabolic profile and whether the effect is influenced by physical activity level or BMI. Knowledge of dietary patterns in the population is important for the prevention and control of CVD and related diseases. Therefore, the aim of this study was to examine whether there were variations in dietary patterns by geographic region, area of residence, and socioeconomic status (SES). We examined the associations of dietary patterns with metabolic risk factors in middle-aged Thais and evaluated whether the associations were modified by physical activity or obesity status.

## 2. Methods

### 2.1. Study Population

Data from the Thai National Health Examination Survey IV (NHES IV) were used. NHES IV was a nationally representative cross-sectional study of the Thai population conducted in 2009. Details of the study design have been described elsewhere [12]. Briefly, a total of 20,426 individuals aged 15 and over, of which 8,582 individuals were aged 30–59 years, were randomly selected using multistage cluster sampling. To rule out the effect of behavioral change due to existing chronic diseases in the present study, we excluded those who were previously diagnosed with diabetes, hypertension, ischemic heart disease, and stroke and those taking lipid lowering medication. Thus, a total of 5,872 adults (2,693 men and 3,179 women) aged 30–59 years were included in the analysis. This study was approved by the Ethical Clearance Committee on Human Rights Related to Research involving Human Subjects, Faculty of Medicine, Ramathibodi Hospital, Mahidol University.

### 2.2. Life Style and Health Data

Data included in the survey were comprised of information about age, education level, area of residence, geographic region, food consumption frequency, smoking status, medical history of diabetes, hypertension, and dyslipidemia, and medication use. Anthropometric measurements including height, weight, and waist circumference were performed using standard procedures. Weight was measured by using a calibrated digital scale, TANITA model HD316, and height was measured by using a stadiometer while standing barefoot with shoulders in a normal position. BMI was calculated as weight in kilograms divided by height squared in meters. Waist circumference was measured at a horizontal plane midway between the iliac crest and lower rib margin in centimeters to the nearest 0.1 cm. Venous blood samples were obtained from participants who had fasted for 12 hours overnight. Blood samples were analyzed to determine fasting plasma glucose, high density lipoprotein cholesterol (HDL-C), and triglyceride (TG) at the central laboratory center in the Faculty of Medicine, Ramathibodi Hospital. Plasma glucose was analyzed by a hexokinase enzyme method. TG was measured by enzymatic colorimetric methods and HDL-C was analyzed by homogeneous enzymatic colorimetric methods using the Hitachi 917 model. Physical activity status was assessed by using the Global Physical Activity Questionnaire (GPAQ) [15]. Leisure time physical inactivity (LTPA) level was assessed and categorized into two groups as high LTPA and low LTPA. High LTPA was defined as having 20 minutes of vigorous intensity activity per day on at least 3 days in a typical week or 30 minutes of moderate-intensity leisure-time activity or 5 days of combination of moderate and vigorous intensity activities at least 600 MET-minutes per week [16]; otherwise LTPA was classified as low. Alcohol consumption was assessed and average daily alcohol intake (gm/day) was calculated according to the WHO guide [17] and then classified into two groups as abstinence to low-risk drinking versus moderate to high risk drinking (cut-off point at >41 gm/day for men and >21 gm/day for women).

#### 2.2.1. Dietary Assessment

Trained interviewers collected participants' food consumption data using a food frequency questionnaire (FFQ) which featured commonly consumed food items. The FFQ was developed and validated during a pilot test [18]. All food items were later categorized into 22 key food groups, formed according to key nutrient component, main food group, culinary use, and risk to chronic diseases in particular CVD (low fat, high fat, fiber, etc.), as shown in Table 6. Trained nurses and interviewers performed a face to face interview using pictures of common food items and a frequency card to facilitate answers. The food groups were as follows: meat, fatty meat, processed meat with high fat, processed meat with high salt, fish, shellfish and squid, animal organ, egg, beans, rice, wheat, glutinous rice, fried food, food with coconut milk, fermented fish/soybean, chili sauce/dip, fruit, milk, soymilk, beverage, bamboo shoot, and vegetables. A pilot test was done in order to test reliability and Cronbach's alpha coefficient of 0.80 was obtained, indicating a relatively acceptable level of interitem reliability for the FFQ.

#### 2.2.2. Dietary Pattern

Dietary patterns were derived from factor analysis using principal component analysis based on 22 food groups from the FFQ. A factor score was created for each individual based on the factor analysis. The factors were analyzed using orthogonal transformed with Varimax options. An eigenvalue of >1.5 and the point in the scree plot where the slope of the curve clearly leveled off indicated the number of factors to be retained. The factor score for each pattern was calculated and assigned to each participant. Three main dietary patterns were labeled based on the food groups for each factor. The three factors with factors loading are shown in Table 1. Pearson's correlation coefficients between scores for the three patterns were very small (*r* < 0.001, all *P* = 0.99), indicating that they had no linear correlation. The scores of each pattern were categorized into quartiles. For SES, a questionnaire regarding household assets was used during the field visit. Consumer items in each participant's household were observed and recorded. The household assets included bed, air conditioner, electric water boiler, washing machine, microwave, personal computer, house telephone, car, and flushing toilet. A SES variable, wealth index score, was calculated based on ownership of household items using factor analysis to assign the indicator weights [19]. The standardized score was assigned to individuals. Wealth quintile was created according to quintile of the scores. The lowest quintile indicated the poorest group and the highest indicated the richest group. To describe smoking habit, each participant was classified as one of the following: nonsmoker, ex-smoker, or current smoker.

### 2.3. Outcome

Metabolic syndrome (MetS) was defined according to the harmonizing criteria [1] as having three or more of the following factors: abdominal obesity (waist circumference ≥80 cm in women and ≥90 cm in men); high blood pressure, systolic blood pressure (SBP) ≥130 mmHg, or diastolic blood pressure (DBP) ≥85 mmHg; low HDL-C <50 mg/dL (1.29 mmol/L) in women and <40 mg/dL (1.04 mmol/L) in men, high triglycerides (TG ≥150 mg/dL (1.69 mmol/L)); hyperglycemia (fasting plasma glucose ≥100 mg/dL (5.6 mmol/L)).

### 2.4. Statistical Analysis

All statistical analyses were weighted to account for the complex survey design. Since dietary pattern is likely to differ by sex, all the statistical tests were performed separately by sex. Means and proportions of participants' metabolic and demographic characteristics, for example, age, sex, urban/rural area, geographic region, and wealth index, were calculated according to dietary pattern quartiles. The Chi-square test was used to compare categorical variables among quartiles. Continuous variables were compared across quartiles using ANOVA. Age-adjusted means of metabolic variables by quartile for each dietary pattern were calculated by using ANCOVA. Multiple logistic regression was used to obtain age-adjusted percentage of each metabolic component (coded as 0 = no, 1 = yes) and MetS prevalence across quartiles. For linear trend analysis, linear regression was used to examine the linear trend of continuous variable of characteristics of subjects across quartile categories for each dietary pattern with each category coded on an ordinal scale. For categorical variables, multivariate logistic regression was used. Multiple logistic regression was used to examine the association of dietary patterns with each metabolic component and MetS, controlling for potential confounding factors including age, family history of diabetes, BMI, and leisure time physical activity. We included BMI as continuous scale variable in the model to further assess whether the effect of dietary pattern was mediated by general obesity. To explore whether the effect of dietary pattern was modified by general obesity or LTPA status, we performed stratified analysis by obesity (at BMI ≥25 and BMI <25 kg/m^2^) or by LTPA status. For obesity status, the results of both obesity strata were similar. We also tested the interaction of dietary patterns with BMI and with LTPA and found no interaction for dietary pattern with BMI, but possible interaction with LTPA (*P* < 0.1 was considered as potential interaction). Consequently, we assessed the association of dietary pattern and MetS stratified by LTPA status. We did not include wealth quintile and geographic region in the multivariable regression, since they were highly correlated with dietary pattern. For women, we also included a variable to further control for menopausal status. Statistical significance level was considered at *P* < 0.05. All the statistical tests were performed using STATA version 10 (Texas, USA).

## 3. Results

Table 1 presents the food groups and factor loadings of 3 dietary patterns labeled as meat, carbohydrate, and healthy patterns. The 3 dietary patterns represent 32.7% of variance explained. The meat pattern explained 11.9% of the variance in men and 12.6% in women; this pattern was characterized by the high factor loadings of meat, fatty meat, processed meat with high fat, processed meat with high salt, shellfish and squid, animal organ, egg, fried food, food with coconut milk, and beverage. The carbohydrate pattern (explained 10.8% and 10.6% of variance in men and women, resp.) included glutinous rice, chili sauce/dip, fermented fish/soybean, and bamboo shoot. The healthy pattern was characterized by a high intake of fish, bean, wheat, fruit, milk, soy milk, and vegetables and explained 10% of the variance in men and 9.9% in women.

Table 2 shows the sociodemographic characteristics of participants in each dietary pattern. Individuals in the highest quartile of intake for the meat pattern were more likely to be younger and resided in the central and southern regions and in Bangkok. The percentage of the meat pattern was different for urban versus rural areas in men, but not in women, as men in urban areas were more likely to be in the fourth quartile compared with men in rural areas. Urban residents were more likely to be in the fourth quartile of the healthy pattern, but less likely to be in the highest quartile of the carbohydrate pattern, in contrast to rural residents. Those in the highest quartile of intake for the carbohydrate pattern were more likely to be residents in rural areas, in the north and northeast regions, whereas those residing in Bangkok were more likely to have a healthy pattern. For socioeconomic status (SES), those in the highest quintile of wealth index were more likely to have the healthy pattern, whereas those in the lower quintile were more likely to have a high carbohydrate intake. Individuals in the highest quartile of the carbohydrate pattern were more likely to be among those with low SES.

Table 3 shows the distribution of metabolic risk factors by dietary patterns. The mean BMI by quartile of meat pattern was relatively similar in both sexes. For the carbohydrate pattern, BMI decreased significantly in the highest quartile in men but was not different in women. Compared with participants in the lowest quartile, both men and women in the highest quartile of the carbohydrate pattern had lower HDL-C levels, higher TG levels, and a higher prevalence of MetS. Both men and women in the highest quartile of the meat pattern had higher levels of HDL-C. Only men in the highest quartile of meat pattern had a higher level of SBP but a lower level of TG compared with those in the lowest quartile. Menopausal status was relatively equally distributed across each dietary pattern.

Table 4 shows the results of multiple logistic regression models for the associations of dietary patterns with each metabolic component and MetS controlling for other covariates. In men, compared with the first quartile, the highest quartile of carbohydrate pattern was associated with increased odds of high TG and low HDL-C, but lower odds of abdominal obesity and hyperglycemia. Meat pattern was associated with increased odds of abdominal obesity, high blood pressure, and hyperglycemia. In women, the meat pattern was associated with increased odds of hyperglycemia. Carbohydrate pattern was likely to be associated with hypertriglyceridemia and low HDL-C, while the healthy pattern was associated with decreased odds of abdominal obesity and hypertriglyceridemia.

The carbohydrate pattern was the only dietary pattern associated with increased odds of MetS in the fourth quartile compared with the first quartile in men (adjusted OR of 1.82, 95% CI 1.31, 2.55) and women (adjusted OR 1.60, 95% CI 1.24, 2.08).

The associations between dietary patterns and MetS as stratified by leisure time physical activity status are shown in Table 5. The odds of metabolic syndrome for the fourth quartile of the carbohydrate pattern remained significantly increased among men and women who were physically inactive (adjusted OR 1.83, 95% CI 1.27, 2.63, 1.70, 95% CI 1.28, 2.27, resp.), but not among those who were physically active, particularly in women.

## 4. Discussion

The important findings in the present study were that the carbohydrate pattern was more popular in the rural areas and in the northeastern and northern region of Thailand, whereas the meat pattern was more common in urban areas, Bangkok, and the central and southern regions. The healthy dietary pattern was also more prevalent in the residents living in urban areas and among those with higher socioeconomic status. Those with the healthy dietary pattern were more likely to have a better metabolic profile. To our knowledge, the present study is the first to report the association of high dietary intake of carbohydrate with glutinous rice with an increased risk of MetS due to high TG and low HDL-C. On the other hand, individuals with a high intake of meat and its product were more likely to have high BP, high FPG, and abdominal obesity. However, the effect of carbohydrate pattern decreased among those with high LTPA and increased among those with low LTPA.

The findings of an association between high carbohydrate intake and increased risk of metabolic risk factors and MetS in the present study were consistent with other studies [5, 8]. A study in Korea reported that risk of MetS increased among people eating white rice compared with those eating rice with beans and rice with multigrains [8]. Kim et al. [5] reported a higher risk of MetS among those in the highest quartile of carbohydrate intake, particularly among those with BMI ≥25 kg/m^2^. However, the present study did not reveal such interaction between BMI and carbohydrate; the effect of the dietary pattern was not different by BMI status. Although it is not clear whether total energy intake is different by pattern, it is very likely that the proportion of energy derived from carbohydrate among those favoring a carbohydrate dietary pattern is greater than in those who favor the other two patterns. A survey of national food insecurity in Thailand reported that people in the northeast had the highest daily consumption of carbohydrate (338.7 gm/person), followed by those in the north at 322.4 gm/person. Daily carbohydrate intake was lowest in Bangkok (268.8 gm/person). But the northeast had the lowest daily intake of energy from fats (51.1 gm/person) compared with a national average of 60.3 gm/person [20]. We found that the healthy pattern including fruit and vegetables, and beans decreased the risk of MetS. MetS has been negatively associated with a healthy diet comprising of fruit and vegetables in several populations [21–23]. A high intake of fruit, vegetables, and fish as part of a healthy diet is associated with a decreased risk of coronary heart disease (CHD) [4, 24] and negatively associated with MetS [25]. The lower risk of MetS among individuals with a healthy diet might be due to higher intakes of dietary fiber, minerals, phytoestrogen, and whole grains [26]. A healthy diet which is high in soy-derived isoflavones decreases the risk of coronary heart diseases and CVD by protecting against dyslipidemia. Clinical research suggests that soy reduces low-density lipoprotein cholesterol (LDL-C) and improves HDL-C and glycemic control [27, 28].

The association of a high meat dietary pattern intake with MetS has been reported in several studies [3, 21, 25]. The Malmo study [20] found an increased risk of hyperglycemia with fatty meat intake in men. Moreover, a significant body of knowledge suggests that high meat intake, especially red meat, is associated with increased risk of CHD and diabetes. The present study found a positive association of meat and its product intake with odds of abdominal obesity, high blood pressure, and hyperglycemia in men, but only with hyperglycemia in women. We did not observe an association between meat pattern and MetS. The difference in the effect of meat pattern from other studies might be due to difference in the data collection tools, amount of meat consumed, and the fat content of the meat consumed. With regard to the benefit of physical activity, the decrease in association of carbohydrate pattern with MetS among physically active individuals found in the present study is consistent with other observational and experimental studies [10, 29, 30]. A population-based study in China reported a lower likelihood of CVD risk factors among those who had both physically active lifestyles and healthy dietary patterns [10]. An increase from moderate to vigorous LTPA was associated with a decreased probability of developing MetS and diabetes in a Finnish randomized controlled study [29]. Menopause might be a risk for MetS; however, in the present study menopause was relatively equally distributed across each dietary quartile and was not significantly associated with MetS. Hence, it was not included in the final model. We included BMI in the model to assess the direct effect of dietary pattern on MetS, not mediated through obesity. However, general obesity was associated with MetS; it is possible that controlling for BMI in the model might have underestimated the effect of diet.

Some limitations in the present study include the shortcoming of factor analysis, as it is not highly reproducible due to some degree of subjective decision. The FFQ used in the present study was not semiquantitative, so only frequency of intakes and not quantities of nutrients consumed could be estimated and quantified as energy intake. The cross-sectional study is a useful design to assess the dietary patterns of people with particular characteristics, but it cannot establish a causal relationship between dietary patterns and health outcomes. Also, in our analysis we did not take into account hormone and supplement use among women; whether this might have confounded the results is not clear and needs further study. Nevertheless, a major strength of the present study is that it involved a nationally representative sample of the Thai population. The implication of this study is that knowledge of regional preferences in dietary patterns allows the design of more specific dietary recommendations for specific groups, such as advice about healthy dietary patterns and more LTPA among those with high consumption of carbohydrate pattern.

## 5. Conclusion 

The difference in dietary patterns by demographic characteristics and geographic areas might contribute to the variation in metabolic profile. The carbohydrate dietary pattern increased the odds of MetS; however, the risk decreased among those with high level of LTPA.

## Figures and Tables

**Table 1:** Factor loading for the first three factors from principal components analysis of food frequency questionnaire interviews, among Thai adults aged 30–59 years, NHES IV, 2009.

Food	Men			Women		
	Meat pattern	Carbohydrate pattern	Healthy pattern	Meat pattern	Carbohydrate pattern	Healthy pattern
Meat	0.60			0.52		
Fatty meat	0.66			0.58		
Processed meat with high fat	0.44			0.48		
Processed meat with high salt	0.45			0.45		
Fish			0.32			0.36
Shellfish and squid	0.40			0.39		
Animal organ	0.47			0.46		
Egg	0.37			0.49		
Beans			0.58			0.59
Rice	0.18			0.25		
Wheat			0.42			0.48
Glutinous rice		0.81			0.79	
Fried food	0.56			0.60		
Food with coconut milk	0.51			0.51		
Fermented fish/soybean		0.81			0.82	
Chili sauce/dip		0.50			0.46	
Fruit			0.61			0.58
Milk			0.56			0.56
Soy milk			0.60			0.61
Beverage	0.36			0.46		
Bamboo shoot		0.48			0.56	
Vegetables			0.29			0.30

Variance explained (%)	11.93	10.81	10.0	12.6	10.6	9.9

Variance in men 32.74; women 33.1.

**Table 2:** Socio-demographic factors by quartile of factor scores of dietary patterns in Thai adults aged 30–59 years, NHES IV, 2009.

	Meat				*P* value	Carbohydrate				*P* value	Healthy				*P* value
	Q1	Q2	Q3	Q4		Q1	Q2	Q3	Q4		Q1	Q2	Q3	Q4	
Men (*n* = 2693)															
Age (mean, yrs)	45.3	44.3	44.1	42.5	<0.001	43.6	44.0	44.0	44.5	0.01	43.5	44.4	43.8	44.6	0.08
Area of residence															
Urban (%)	25.9	23.9	22.4	27.9	0.02	33.2	25.5	24.7	16.5	<0.001	23.1	21.0	24.7	31.2	<0.001
Rural (%)	27.3	25.8	24.7	22.3		13.6	19.7	24.6	42.0		27.4	29.9	22.9	19.8	
Region															
North (%)	28.0	28.6	26.7	16.6	<0.001	5.7	17.8	34.5	41.9	<0.001	24.4	30.4	25.0	20.1	<0.001
Central (%)	20.6	21.6	26.0	31.9		31.9	38.2	24.8	5.1		25.5	25.5	22.9	26.1	
Northeast (%)	33.3	26.1	21.2	19.4		2.4	8.5	23.7	65.4		28.6	30.9	21.7	18.9	
South (%)	16.3	25.6	27.3	30.8		52.3	33.3	12.4	2.0		26.2	23.1	25.6	25.0	
Bangkok (%)	25.4	23.3	20.9	30.3		48.9	26.9	21.1	3.1		22.1	15.5	25.7	36.7	
Wealth quintile															
Q1 (%)	31.8	25.3	23.8	19.1	0.01	10.4	17.3	24.2	48.1	<0.001	32.4	32.6	19.8	15.1	<0.001
Q2 (%)	26.1	28.2	24.5	21.1		12.0	18.8	26.7	42.5		26.7	32.6	24.0	16.7	
Q3 (%)	24.3	23.1	24.9	27.6		20.5	19.0	25.5	34.9		26.1	26.5	25.1	21.9	
Q4 (%)	24.0	23.3	25.2	27.6		22.4	27.6	24.0	26.0		24.5	22.2	23.1	30.2	
Q5 (%)	27.2	26.1	21.6	25.1		34.0	26.7	22.0	17.2		18.8	19.9	21.1	36.1	
Women (*n* = 3179)															
Age (yr)	44.7	44.3	43.6	41.9	<0.001	43.4	43.6	43.8	43.7	0.51	43.8	43.2	43.9	43.6	0.95
Area of residence															
Urban (%)	26.8	23.2	22.3	27.8	0.08	31.7	26.7	23.7	17.8	<0.001	23.5	21.3	26.0	29.1	<0.001
Rural (%)	26.4	25.8	24.3	23.4		14.5	17.7	25.3	42.6		28.6	27.8	24.3	19.3	
Region															
North (%)	29.8	25.2	25.6	19.4	<0.001	6.5	15.0	29.1	49.4	<0.001	25.5	29.8	27.5	17.2	<0.001
Central (%)	20.7	23.4	25.7	30.2		34.2	31.2	29.4	5.2		31.4	21.1	23.2	24.3	
Northeast (%)	33.6	27.2	19.8	19.3		2.6	6.6	23.8	67.0		26.4	29.8	23.9	19.9	
South (%)	15.1	24.5	31.6	28.8		44.9	39.4	13.2	2.5		24.9	23.6	23.8	27.6	
Bangkok (%)	22.2	21.4	19.0	37.4		41.6	31.7	22.3	4.4		24.8	17.7	27.9	29.6	
Wealth quintile															
Q1 (%)	32.4	26.5	20.2	20.9	<0.001	8.9	12.6	25.8	52.6	<0.001	33.3	34.2	23.1	9.5	<0.001
Q2 (%)	23.5	26.3	25.4	24.9		12.3	17.9	26.7	43.1		33.3	26.8	24.1	15.8	
Q3 (%)	21.8	26.9	26.1	25.3		17.3	24.2	24.6	33.9		28.4	28.7	22.5	20.3	
Q4 (%)	23.2	22.5	26.2	28.1		23.8	24.4	23.2	28.5		26.6	19.6	26.4	27.4	
Q5 (%)	31.0	22.9	21.3	24.7		34.0	22.3	23.9	19.8		15.2	19.4	28.1	37.2	

Data are shown in means and percent.

**Table 3:** Age-adjusted means and percentage of metabolic risk factors by dietary pattern in Thai adults aged 30–59 years, NHES IV, 2009.

	Meat				*P* for trend	Carbohydrate				*P* for trend	Healthy				*P* for trend
	Q1	Q2	Q3	Q4		Q1	Q2	Q3	Q4		Q1	Q2	Q3	Q4	
Men (*n* = 2693)															
BMI (kg/m^2^)	23.1	23.1	23.2	23.5	0.08	23.6	23.5	23.0	23.0	<0.01	23.2	22.9	23.3	23.6	0.01
WC (cm)	79.5	79.1	80.3	80.9	<0.01	81.2	81.4	79.4	78.8	<0.001	80.1	78.9	80.0	81.1	0.02
SBP (mm Hg)	120.6	119.8	121.9	124.0	0.001	123.4	122.0	121.0	120.7	0.01	121.1	120.3	122.5	122.5	0.20
HDL-C (mg/dL)	43.8	45.3	45.5	45.4	0.03	46.7	46.2	46.0	42.7	<0.001	45.3	44.5	44.3	46.0	0.39
Triglyceride (mg/dL)	194.3	169.6	172.3	173.1	0.005	154.9	180.5	170.8	193.8	0.001	176.1	179.5	186.9	168.3	0.42
FPG (mg/dL)	85.7	87.6	88.0	88.8	<0.01	90.5	91.5	88.4	82.8	<0.001	86.4	87.8	88.0	87.8	0.04
MetS (%)	18.8	16.7	18.5	21.5	0.10	17.3	20.7	17.5	19.4	0.65	18.2	17.8	20.5	19.0	0.37
Abdominal obesity (%)	15.0	13.5	19.0	18.7	<0.01	21.0	19.3	16.4	12.4	<0.001	17.6	13.7	15.5	19.4	0.25
High TG (%)	49.7	43.2	46.1	47.2	0.57	35.4	42.4	46.2	55.2	<0.001	43.0	48.9	52.2	42.2	0.78
Low HDL-C (%)	39.9	35.6	33.6	33.5	0.02	29.8	31.8	28.7	46.2	<0.001	34.3	37.5	39.1	32.0	0.46
High BP (%)	29.3	25..5	28.8	39.2	0.001	35.5	33.0	27.3	28.7	0.01	30.3	27.4	34.8	30.3	0.35
Hyperglycemia (%)	10.4	14.7	16.4	17.2	0.001	17.7	17.2	16.8	9.7	<0.001	13.5	14.4	15.1	15.0	0.36
Alcohol drinker	10.3	13.0	14.2	16.8	<0.01	13.9	14.3	16.4	10.7	0.16	15.1	13.3	14.2	11.1	0.04
Low LTPA (%)	75.7	82.4	76.2	80.0	0.47	79.1	75.0	77.9	80.8	0.16	84.9	81.7	77.0	69.0	<0.001
Women (*n* = 3179)															
BMI (kg/m^2^)	24.6	25.1	24.7	25.0	0.20	24.7	25.2	24.9	24.7	0.39	25.1	25.1	24.6	24.5	0.001
WC (cm)	78.4	79.5	79.0	79.7	0.04	78.8	79.4	79.6	78.9	0.96	79.9	79.3	78.9	78.3	<0.01
SBP (mm Hg)	117.9	118.4	118.4	117.7	0.79	117.2	118.6	118.3	118.2	0.32	119.1	118.5	117.4	117.3	0.001
HDL-C (mg/dL)	48.5	48.4	49.9	51.1	<0.001	51.3	51.3	50.4	46.7	<0.001	49.2	49.6	48.6	50.4	0.10
Triglyceride (mg/dL)	134.9	133.8	134.5	124.0	0.05	119.9	122.0	126.4	148.0	0.001	133.1	131..9	133.9	128.0	0.19
FPG (mg/dL)	83.6	86.4	85.5	85.9	0.04	88.3	86.8	85.1	83.0	<0.01	85.4	85.4	85.3	85.2	0.74
MetS (%)	23.4	25.6	24.1	22.5	0.62	22.1	21.4	22.3	27.5	0.02	28.0	22.8	22.5	21.8	0.01
Abdominal obesity (%)	43.4	44.0	42.5	46.4	0.36	43.5	43.5	45.6	44.2	0.51	50.0	43.7	43.1	39.6	<0.001
High TG (%)	27.8	31.8	29.1	28.1	0.87	22.8	23.2	27.2	37.6	<0.001	30.6	29.2	31.4	25.1	0.03
Low HDL-C (%)	57.0	61.5	55.0	50.7	<0.01	50.1	51.2	50.3	66.3	<0.001	56.5	55.6	58.1	54.1	0.49
High BP (%)	22.1	23.1	24.8	22.8	0.61	23.7	25.4	24.9	20.4	0.09	25.5	23.4	19.8	23.8	0.19
Hyperglycemia (%)	6.6	9.9	10.2	12.2	0.001	13.3	9.5	8.7	8.1	0.021	11.4	8.8	8.2	9.6	0.29
Alcohol drinker (%)	0.5	1.1	1.7	3.6	<0.001	0.9	0.7	3.0	1.8	0.02	2.3	2.2	1.3	0.9	<0.01
Low LTPA (%)	79.9	84.7	82.0	83.3	0.21	83.3	83.7	82.1	81.5	0.34	90.5	82.9	81.1	73.6	<0.001
Menopause (%)	23.3	22.0	22.6	23.0	0.88	23.6	22.3	23.9	21.6	0.27	21.7	23.1	23.1	23.1	0.38

BMI: body mass index. WC: waist circumference. High TG: high triglycerides. HDL-C: high density-lipoprotein cholesterol. FPG: fasting plasma glucose. SBP: systolic blood pressure. LTPA: leisure time physical activity. Alcohol drinker: consumed alcohol >41 gm/day for men and >21 gm/day for women.

**Table 4:** Adjusted odds ratio (95% CI) for metabolic syndrome and its components according to quartile of factor scores of dietary patterns in Thai adults aged 30–59 years, NHES IV, 2009.

	Abdominal obesity	High TG	Low HDL-C	High BP	Hyperglycemia	MetS
Men (2693)						
Age	1.02 (1.01, 1.03)	1.00 (0.99, 1.01)	1.01 (1.00, 1.02)	1.04 (1.03, 1.05)	1.05 (1.04, 1.06)	1.05 (1.04, 1.07)
Meat						
Q1	1	1	1	1	1	1
Q2	0.93 (0.70, 1.23)	0.75 (0.62, 0.91)	0.83 (0.66, 1.03)	0.81 (0.63, 1.04)	1.55 (1.19, 2.01)	0.82 (0.61, 1.10)
Q3	1.45 (1.10, 1.92)	0.87 (0.69, 1.11)	0.78 (0.64, 0.95)	0.99 (0.77, 1.25)	1.74 (1.33, 2.27)	0.89 (0.63, 1.25)
Q4	1.40 (1.10, 1.83)	0.92 (0.74, 1.14)	0.80 (0.64, 0.99)	1.56 (1.24, 1.97)	1.78 (1.32, 2.41)	1.01 (0.82, 1.23)
Carbohydrate						
Q1	1	1	1	1	1	1
Q2	0.94 (0.69, 1.28)	1.32 (1.08, 1.60)	1.08 (0.87, 1.35)	0.90 (0.72, 1.12)	0.93 (0.690, 1.25)	1.34 (1.00, 1.80)
Q3	0.79 (0.57, 1.07)	1.58 (1.26, 1.98)	0.94 (0.75, 1.19)	0.68 (0.54, 0.85)	0.94 (0.66, 1.35)	1.40 (1.07, 1.84)
Q4	0.60 (0.46, 0.79)	2.26 (1.69, 3.03)	1.93 (1.50, 2.47)	0.79 (0.62, 1.00)	0.52 (0.36, 0.75)	1.82 (1.31, 2.55)
Healthy						
Q1	1	1	1	1	1	1
Q2	0.79 (0.59, 1.07)	1.18 (0.94, 1.48)	1.05 (0.87, 1.26)	0.90 (0.69, 1.18)	1.13 (0.82, 1.54)	1.09 (0.79, 1.51)
Q3	0.83 (0.63, 1.10)	1.40 (1.16, 1.69)	1.20 (1.00, 1.43)	1.19 (0.93, 1.52)	1.05 (0.77, 1.42)	1.11 (0.76, 1.61)
Q4	1.03 (0.79, 1.35)	0.92 (0.74, 1.15)	0.88 (0.75, 1.02)	0.91 (0.71, 1.17)	1.03 (0.76, 1.39)	0.91 (0.67, 1.23)
Women (3179)						
Age	1.03 (1.02, 1.04)	1.04 (1.03, 1.05)	0.99 (0.98, 1.00)	1.08 (1.07, 1.09)	1.08 (1.06, 1.10)	1.07 (1.05, 1.08)
Meat						
Q1	1	1	1	1	1	1
Q2	1.05 (0.82, 1.34)	1.31 (1.03, 1.66)	1.28 (1.04, 1.58)	1.06 (0.82, 1.39)	1.65 (1.07, 2.53)	1.07 (0.84, 1.36)
Q3	0.99 (0.83, 1.19)	1.21 (0.94, 1.56)	1.02 (0.82, 1.26)	1.17 (0.86, 1.61)	1.62 (1.19, 2.20)	1.13 (0.84, 1.52)
Q4	1.17 (0.98, 1.41)	1.01 (0.88, 1.49)	0.86 (0.70, 1.05)	1.03 (0.79, 1.35)	1.96 (1.30, 2.96)	0.94 (0.72, 1.21)
Carbohydrate						
Q1	1	1	1	1	1	1
Q2	1.03 (0.85, 1.27)	1.01 (0.81, 1.26)	1.03 (0.84, 1.27 )	1.09 (0.90, 1.32)	0.66 (0.48, 0.92)	0.86 (0.65, 1.14)
Q3	1.20 (1.00, 1.45)	1.31 (1.07, 2.77)	1.04 (0.83, 1.30)	1.11 (0.89, 1.40)	0.65 (0.48, 0.89)	1.02 (0.82, 1.28)
Q4	1.11 (0.88, 1.40)	2.12 (1.62, 2.77)	2.03 (1.63, 2.54)	0.85 (0.66, 1.09)	0.63 (0.39, 1.02)	1.60 (1.24, 2.08)
Healthy						
Q1	1	1	1	1	1	1
Q2	0.81 (0.65, 1.01)	0.84 (0.56, 1.09)	0.92 (0.78, 1.09)	0.89 (0.74, 1.09)	0.83 (0.55, 1.24)	0.64 (0.50, 0.81)
Q3	0.79 (0.70, 0.94)	0.93 (0.73, 1.19)	1.02 (0.88, 1.18)	0.72 (0.57, 0.90)	0.73 (0.46, 1.17)	0.69 (0.523, 0.91)
Q4	0.67 (0.57, 0.79)	0.74 (0.59, 0.92)	0.97 (0.82, 1.15)	0.89 (0.67, 1.18)	0.86 (0.54, 1.37)	0.72 (0.52, 0.99)

High TG: high triglycerides. Low HDL-C: low high density-lipoprotein cholesterol. High BP: high blood pressure. MetS: metabolic syndrome.

All models were controlled for age, alcohol drinking, family history of diabetes and smoking, leisure time physical activity, and BMI.

**Table 5:** Adjusted odds ratio (95% CI) for metabolic syndrome according to quartile of factor scores of dietary patterns by leisure time physical activity status in Thai adults aged 30–59 years, NHES IV, 2009.

	Men		Women	
	Low LTPA (*n* = 2075)	High LTPA (*n* = 618)	Low LTPA (*n* = 2600)	High LTPA (*n* = 579)
Age	1.06 (1.04, 1.08)	1.04 (1.01, 1.07)	1.06 (1.04, 1.08)	1.11 (1.08, 1.14)
Meat				
Q1	1	1	1	1
Q2	0.81 (0.54, 1.22)	0.78 (0.32, 1.91)	1.07 (0.84, 1.35)	1.00 (0.58, 1.72)
Q3	0.75 (0.52, 1.09)	1.57 (0.83, 2.98)	0.99 (0.70, 1.42)	1.40 (0.87, 2.27)
Q4	1.02 (0.75, 1.39)	0.84 (0.33, 2.09)	0.84 (0.61, 1.16)	0.90 (0.38, 2.17)
Carbohydrate				
Q1	1	1	1	1
Q2	1.37 (0.99, 1.90)	1.38 (0.64, 2.98)	0.84 (0.64, 1.10)	0.90 (0.38, 2.17)
Q3	1.37 (1.00, 1.87)	1.58 (0.72, 3.44)	0.98 (0.72, 1.32)	1.08 (0.49, 2.35)
Q4	1.83 (1.27, 2.63)	1.88 (0.96, 3.68)	1.70 (1.28, 2.27)	1.08 (0.53, 2.22)
Healthy				
Q1	1	1	1	1
Q2	1.06 (0.80, 1.41)	1.17 (0.41, 3.30)	0.58 (0.44, 0.76)	0.97 (0.42, 2.24)
Q3	1.13 (0.74, 1.72)	0.82 (0.32, 2.14)	0.62 (0.45, 0.87)	1.28 (0.56, 2.91)
Q4	0.87 (0.64, 1.19)	0.89 (0.42, 1.90)	0.71 (0.49, 1.05)	0.84 (0.37, 1.93)

LTPA: leisure-time physical activity.

Model included age, family history of diabetes, smoking, alcohol drinking, and BMI.

**Table 6:** Food groups and food items from the food frequency questionnaire.

Food groups	Food items
Meat	Lean pork/beef, chicken/duck without skin
Fatty meat	Meat with fat, chicken/duck with skin, streaky pork
Processed meat with high fat	Processed meat high in fat, that is, sausage, Thai style sausage, Chinese sausage, sour sausage, bacon, ham, Vietnamese ham
Processed meat with high salt	Processed meat high in salt, that is, meat floss, salted fish, salted sun-dried beef/pork/fish
Fish	Fresh-water fish, salt-water fish, and so forth
Shellfish and squid	Crustacean and mollusk seafood, that is, shrimp, crab, squid, clam, and so forth
Animal organ	Liver, blood jelly, intestine, gizzard, and so forth
Egg	Egg
Beans	Beans and its products, that is, mung bean, soybean, peanut, tofu, Kaset protein
Rice	Polished rice
Wheat	Whole wheat, whole grain bread
Glutinous rice	Sticky rice
Fried food	Fried pork, fried chicken, friend banana, friend potato, fried meat ball, fish cake, and so forth
Food with coconut milk	Any dishes cooked with coconut milk, that is, spicy curry with coconut milk, green beef curry with coconut milk, and so forth
Fermented fish/soybean	Condiment of fermented fish, southern style fish sauce, Thai style fermented soybean, and so forth
Chili sauce/dip	Thai dipping sauce, that is, roasted chili paste, shrimp paste sauce, green chili dip, fermented fish spicy dip
Fruit	Fresh fruits, that is, orange, banana, guava, ripe papaya, pineapple, longan, watermelon, sugar apple, grapes, rambutan
Milk	Milk
Soy milk	Soy milk
Beverage	Soda beverage
Bamboo shoots	Bamboo shoots
Vegetables	Chinese kale, cauliflower, cabbage, ivy gourd, sponge gourd, Thai water morning glory, green onion
